# Score reliability and factor similarity of the Sociocultural Attitudes Towards Appearance Questionnaire-3 (SATAQ-3) among four ethnic groups

**DOI:** 10.1186/2050-2974-1-14

**Published:** 2013-04-22

**Authors:** Cortney S Warren, David H Gleaves, Liya M Rakhkovskaya

**Affiliations:** 1 Department of Psychology, University of Nevada, Las Vegas. 4505 Maryland Parkway, Las Vegas, NV 89154, USA; 2 University of South Australia, Fenn Pl, Adelaide, SA 5000, Australia

**Keywords:** Sociocultural Attitudes Towards Appearance Questionnaire (SATAQ-3), Media pressure, Thin-ideal internalization, Ethnicity, Psychometric equivalence

## Abstract

**Background:**

This study evaluated the score reliability and equivalence of factor structure of the Sociocultural Attitudes towards Appearance Questionnaire-3 (SATAQ-3) [1] in a sample of female college students from the four largest ethnic groups in the USA.

**Methods:**

Participants were 1245 women who self-identified as European American/White (*n* = 543), African American/Black (*n* = 137), Asian American (*n* = 317), or Latina/Hispanic (*n* = 248). All completed the SATAQ-3 and a demographic questionnaire. To test the factor similarity and score reliability across groups, we used exploratory factor analysis and calculated Cronbach’s alphas (respectively).

**Results:**

Score reliability was high for all groups. Tests of factor equivalence suggested that the four pre-established factors of the SATAQ-3 (i.e., knowledge, perceived pressure, thin-ideal internalization, athletic-ideal internalization) were similar for women of all ethnic groups. Only two items (20 and 27) did not consistently load on the previously identified scale across all four groups. When scored, African Americans reported significantly less perceived pressure and internalization than all other groups.

**Conclusions:**

Results support the use of the SATAQ-3 in female college students of these four ethnicities.

## Background

Images of the ideal-looking woman inundate mainstream Western media [2]. Through film, advertisements, television shows, music videos, and magazines, the ideal woman in Western cultures (e.g., in mainstream USA, Australia, Western Europe) is depicted as thin, young, tall, and fit with long hair, a thin waist and fair skin [3,4]. For example, Spitzer, Henderson and Zivian [4] found that nearly all *Playboy* and *Miss America* pageant contestants from 1977 to 1996 were underweight, with 17-33% meeting diagnostic weight criteria for anorexia nervosa. In a similar study, Voracek and Fisher [5] found that *Playboy* centerfold models’ bust size, hip size, and waist-to-hip ratio decreased significantly from 1950’s to the 1990’s.

In addition to idealizing and valuing a specific physique, mainstream Western culture and media promote the message that attaining the ideal appearance assures social status, happiness, success, and personal worth for women [6-8]. For example, Stokes and Frederick-Recascino [8] found significant positive correlations between happiness and perceptions of sexual desirability, weight satisfaction, and physical fitness among adult women. Furthermore, Western cultures’ emphasis on individualism, self-reliance, and competitiveness makes women responsible for maintaining a low weight and an ‘attractive’ physical appearance [6,9-11]. Combined, cultural values and ideals of appearance promoted by mainstream Western media emphasize that attaining a thin, fit body is fundamental to women’s social value and moral character.

### Media exposure and eating pathology

Conceptually, values and ideals of appearance promoted in mainstream Western media are etiologically tied to the development of eating pathology through a progression of knowledge, perceived pressure, and internalization [1,12,13]. As women are exposed to Western media, they develop increased *information* or *awareness* of cultural values and ideals of appearance. As such awareness increases, women often report increased *pressure* to attain the ideal and *internalize* these ideals by adopting them as self-relevant and behaviorally try to attain them (e.g., through dieting, exercise, make-up). For women, internalization often manifests in a desire to look like very thin or fit models in the media (i.e., *thin-* and *athletic-ideal internalization,* respectively).

A growing body of research supports this model and the role of internalization of Western sociocultural values and ideals of appearance on eating pathology in women [12,14-20]. In a longitudinal study of 216 children ages 6–14, thin-ideal internalization prospectively predicted increased body dissatisfaction, depressive symptoms, preoccupation with thinness, and a larger perceived body size for girls [21]. Similarly, a meta-analysis of cross-sectional data examining risk and maintenance factors for eating pathology found a medium to large association between thin-ideal internalization and body dissatisfaction in women [22]. Although less studied to date, research also suggests that athletic-ideal internalization is associated with increased compulsive exercise [23] and body dissatisfaction [24,25] in women.

### Measurement of sociocultural attitudes towards appearance and ethnicity

Given the strong relationship between internalization of mainstream Western media ideals and eating pathology [12-17,21,22,24-26], it is critical for researchers and clinicians to have psychometrically sound assessment tools measuring these constructs. The Sociocultural Attitudes Towards Appearance Questionnaire-3 (SATAQ-3) [1] is one of the most commonly used self-report measures of endorsement of Western appearance ideals. A 30-item questionnaire with four subscales that correspond with the theoretical model described above (i.e., information, perceived pressure, thin- and athletic-ideal internalization), the SATAQ-3 has been used in many populations including patients with eating disorders [27,28], adolescents [29-32], college students [33,34], and community samples [35-37].

Despite the widespread use of the SATAQ-3, data on its administration to different racial/ethnic groups in the USA are sparse. Recently, Franko and colleagues [38] found that the SATAQ-3 demonstrated adequate score reliability and validity among Latina college students. However, few data exist on the psychometric properties of the SATAQ-3 among African American/Black and Asian American female college students. This is problematic because research indicates considerable ethnic differences in appearance ideals and eating pathology [39-42]. For example, in a meta-analysis of body dissatisfaction among European American/White, African American/Black, Asian American, and Latina women, Grabe and Hyde [40] found that European American/White women reported higher body dissatisfaction than African American/Black women. Nouri, Hill, and Orrell-Valente [43] found similar levels of thin-ideal internalization and body dissatisfaction among European American and Asian American college females. However, a study examining body dissatisfaction among Asian subgroups [44] found that Chinese females reported low body- and self-dissatisfaction, whereas Japanese females reported high body- and self-dissatisfaction (despite similarly low body size in both groups). As such, the relationship between ethnicity and endorsement of Western media is complex and may influence the utility of the SATAQ-3 in various ethnic groups.

### Current study

Given the importance of studying the influence of Western cultural ideals promoted by the media on eating pathology in different cultural and ethnic samples, this study evaluated the score reliability and equivalence of factor structure of the SATAQ-3 in a sample of European American/White, African American/Black, Asian American, and Hispanic/Latina female college students in the USA. Consistent with previous research, we predicted that the SATAQ-3 would have good psychometric properties in female college students of all ethnic groups.

## Method

### Participants

Data from 1245 American female college students in the southwest USA were selected for this study from a large ongoing study investigating eating pathology in female college students. Women were recruited from introductory psychology classes between 2010 and 2012 and all received research credit in exchange for study participation. Although any woman could participate in the larger study, only women who self-identified as a member of one of the four ethnic groups of interest (*N* = 1347 women) with no missing data were included in this study (which excluded 102 participants). This yielded a total sample of European American/White (*n* = 543; 43.6%), African American/Black (*n* = 137; 11.0%), Asian American (*n* = 317; 25.5%), or Latina (*n* = 248; 19.9%) women with complete data.

### Measures

#### Demographics

Participants reported their gender, age, weight, height, ethnicity, and generational status.

#### Sociocultural attitudes toward appearance

As discussed above, the SATAQ-3 [1] is a 30-item measure of endorsement of Western sociocultural beauty standards depicted in the media. Items are rated on a five point response scale ranging from *definitely disagree* (1) to *definitely agree* (5), with higher scores indicating greater media endorsement. According to the original validation by Thompson and colleagues [1], the SATAQ-3 has four factors/subscales: Information (INFO), Perceived Pressure (PRESS), Internalization-General (INT-GEN), and Internalization-Athlete (INT-ATH). Specifically, INFO consists of 9 items measuring recognition of the social importance of the media’s messages about Western beauty ideals (e.g., “Magazine advertisements are an important source of information about fashion and "being attractive”); PRESS consists of 7 items assessing perceived feelings of pressure to conform to the Western ideals exhibited by the media (e.g., “I've felt pressure from TV or magazines to have a perfect body”); INT-GEN consists of 9 items measuring internalization of the thin-ideal (e.g., “I compare my body to the bodies of people who are on TV” and “I wish I looked like the models in music videos”); and INT-ATH consists of 5 items measuring internalization of the athletic ideal (e.g., "I compare my body to that of people in "good shape" and “I try to look like sports athletes”). Previous research suggests that the SATAQ-3 has high internal consistency in female undergraduates [1] and patients with eating disorders [27].

#### Statistical analyses

For all statistical analyses, we used IBM SPSS Statistics version 19. In addition to examining descriptive information, we calculated Cronbach’s alphas for the total score and each subscale score based on the factor structure identified by Thompson and colleagues [1] to measure score reliability. To examine the factor structure of the SATAQ-3, we first examined the Kaiser-Meyer-Olkin measure of sampling adequacy (MSA) [45] to ensure that the data were appropriate for factor analysis. According to Kaiser, MSA values in the .80 - .90 range or higher are ideal and values greater than .70 are adequate. We then conducted an exploratory factor analysis with principal axis extraction (as originally done by Thompson et al. [1]) and oblimen (oblique) rotation separately for each group. We specified a four-factor model because of the established structure of the SATAQ-3, but also examined how well that fit the data.

We then tested the similarity of the factor structure across the four groups using Tucker’s congruence coefficient [46], which is one way to examine factor similarity of measures with complex structure across different samples [47,48]. Llorente, Warren, Lourdes Pérez de Eulate and Gleaves [49] and Wilksch and Wade [50] recently used this method to examine the equivalence of the factor structure the SATAQ-3 across different groups. Although confirmatory factor analysis is sometimes used in such situations, Lorenzo-Seva and ten Burge [46] noted that “exploratory factor analysis (EFA) might be more appropriate as a basis for factor comparisons than the CFA approach in most applications, especially in large multidimensional solutions that do not approach very simple structures.” Lorenzo-Seva and ten Burge [46] found that values for congruence coefficients ranging from .85–.94 indicate fair similarity and values higher than .95 indicate that two factors can be considered equal.

## Results

### Descriptive analyses

As shown in Table 1, participants averaged about 21 years of age (range = 18 to 64) and were in the average weight range [51]. ANOVA’s indicated that ethnic groups differed significantly on age [*F* (3, 1201) = 7.42, *p* < .01] and BMI [*F* (3, 1221) = 9.63, *p* < .01] such that Asian Americans were significantly younger than both European Americans and African Americans; and Asian Americans had a significantly lower BMI than all other groups.

**Table 1:** Means (SDs) and ANOVAs examining demographic and outcome variables by ethnic group

**Variable**	**European Am**	**African Am**	**Latina**	**Asian Am**	***F***	***p***	***η***^**2**^
Age	21.30 (5.91)a	21.21 (5.44)a	20.60 (4.89)	19.65 (2.80)b	7.42	<.01	.02
BMI	23.60 (5.09)a	24.73 (6.46)a	23.88 (5.48)a	22.24 (3.75)b	9.63	<.01	.02
INFO	25.97 (10.16)a	26.99 (10.19)	26.68 (10.20)	28.59 (9.81)b	4.54	<.01	.01
PRESS	22.23 (8.79)a	17.37 (9.20)b	20.72 (8.93)a	20.84 (8.48)a	11.44	<.01	.03
INT-GEN	30.28 (10.34)a	22.44 (10.89)b	27.88 (10.73)c	28.89 (10.24)ac	21.04	<.01	.05
INT-ATH	17.07 (5.11)a	13.56 (6.04)b	15.77 (5.28)c	15.75 (5.19)c	17.52	<.01	.04

*Note.* Means in the same row that do not share subscripts differ at *p* < .05 on post hoc tests with Bonferroni correction. *BMI* = Body Mass Index; *INFO* = Information; *PRESS* = Perceived Pressure; *INT-GEN* = Thin-ideal Internalization; *INT-ATH* = Athletic-ideal Internalization.

There were statistically significant ethnic differences on all of the SATAQ-3 subscales when examined according to the original scoring by Thompson and colleagues [1]: INFO, *F* (3, 1241) = 4.54, *p* < .01; PRESS, *F* (3, 1241) = 11.44, *p* < .01; INT-GEN, *F* (3, 1241) = 21.04, *p* < .01; INT-ATH, *F* (3, 1241) = 17.52, *p* < .01. As displayed in Table 1, Asian Americans reported significantly more knowledge of Western media ideals (INFO) than European Americans; African Americans reported significantly less perceived pressure (PRESS) and thin-ideal internalization (INT-GEN) than all other groups; European Americans reported significantly more thin-ideal internalization than African Americans and Latinas; European Americans reported more athletic-ideal internalization (INT-ATH) than all other groups; and, African Americans reported less athletic-ideal internalization than all other groups.

### Internal consistency and sampling adequacy

In all four groups, score reliability was high and generally equivalent: Cronbach’s alphas for the total score were .97 for each of the four groups. When examined by subscale, INFO, PRESS, and INT-GEN were above .95 and INT-ATH was above .89 for all ethnic groups (see Table 2). MSAs by group were also very similar and good (MSA = .96 for European Americans; .93 for African Americans; .96 for Latinas; and .96 for Asian Americans). We also examined MSAs for individual items to see whether there were any problematic items in any of the groups: All were above .90 for European Americans, Asian Americans, and Latinas and .86 for African Americans. Thus, all of the datasets were clearly suitable for factor analysis.

**Table 2:** Congruence coefficients and Cronbach’s alpha values (α) by subscale and ethnicity

**Subscale**	**Ethnicity**	**1**	**2**	**3**	**4**	**α**
INFO						
	1. European Am	-	.99*	.99*	.99*	.97
	2. African Am		-	.98	.98	.96
	3. Latina			-	.99*	.96
	4. Asian Am				-	.96
PRESS						
	1. European Am	-	.96	.98	.98	.96
	2. African Am		-	96	.96	.98
	3. Latina			-	.98	.96
	4. Asian Am				-	.95
INT-GEN						
	1. European Am	-	.96	.98	.96	.96
	2. African Am		-	.95	.91	.96
	3. Latina			-	.97	.96
	4. Asian Am				-	.96
INT-ATH						
	1. European Am	-	.99*	.98	.99*	.89
	2. African Am		-	99	.98	.99*
	3. Latina			-	.98	.89
	4. Asian Am				-	.90

*Note.* Congruence coefficients are based on factor analysis results whereas the alphas are based on traditional scoring by Thompson and colleagues (2004).

### Examination of factor structures

For the factor analyses, we specified a four-factor model because of the previously identified factor structure by Thompson and colleagues [1]. However, we also examined the degree to which a four-factor model seemed appropriate. For European Americans, there were four factors with initial eigenvalues greater than 1.0 followed by a clear drop-off; thus a four-factor solution appeared appropriate, which explained 74.49% of the total variance. For African Americans, there were three factors with eigenvalues of greater than 1.0; however the fourth was just below 1.0 (0.96) and there was a more clear drop-off after that. Thus, a four-factor solution, which explained 74.66% of the total variance, also seemed appropriate for this group. For Latinas, there were four factors that accounted for 74.54% of the total variance, and there was a clear drop-off between the fourth and fifth factors; thus, four factors again seemed appropriate. Finally, for Asian Americans, there were four eigenvalues greater than 1.0 and a clear drop-off after that. The four extracted factors explained 72.89% of the total variance.

Rotated factor loadings (from the factor pattern matrix) by ethnic group and previously identified subscale (i.e., INFO, PRESS, INT-GEN, and INT-ATH) are depicted in Table 3. Factor loadings of .40 or greater, which we used as the minimum criterion for determining if an item loaded on a particular factor, appear in bold. We used .40 because it was the minimum studied in Guadagnoli and Velicer’s 1988 study [52] of the relationship between sample size and factor stability. In most instances, the four previously identified factors were clearly evident and matched the four subscales identified by previous research. However, the factors were not always in the same order. For European Americans and Latinas, Factor 1 was clearly INT-GEN; Factor 2 was INFO; Factor 3 was INT-ATH; and Factor 4 was PRESS. For Asian and African Americans, factors 1 and 4 switched places, whereas 2 and 3 were the same as European and Latinas. Only two items (20 and 27) did not consistently load on the previously identified scale across all four groups.

**Table 3:** Rotated factor loadings by ethnic group and subscale

***ORIGINAL SUBSCALE***	**European American**				**Latina American**				**Asian American**				**African American**			
	**Factor**				**Factor**				**Factor**				**Factor**			
Item	1	2	3	4	1	2	3	4	1	2	3	4	1	2	3	4
*INFO*																
1	-.03	.**85**	-.02	-.04	-.07	**.91**	-.01	-.06	.05	**.73**	-.08	-.05	.06	**.82**	.02	.13
5	-.05	.**85**	-.00	.08	-.03	**.87**	.05	-.06	.01	**.77**	-.04	-.05	.07	**.82**	.02	.10
9	.02	.**82**	.05	-.07	.07	**.78**	.03	.00	-.06	**.83**	.07	-.04	-.09	**.79**	.02	-.15
13	.05	.**88**	-.04	-.06	.06	**.81**	.02	.00	-.06	**.92**	.03	.03	-.09	**.74**	-.06	-.18
17	-.04	.**92**	.00	.03	.09	**.84**	-.10	.05	.01	**.93**	-.04	.04	-.07	**.88**	-.04	-.10
21	.00	.**89**	.03	-.02	.01	**.92**	-.03	-.00	.09	**.88**	-.03	.07	-.05	**.90**	-.05	-.08
25	.06	.**86**	-.01	.01	.01	**.81**	.06	.07	.04	**.86**	.01	-.01	.13	**.88**	-.03	.05
28	.04	.**83**	.02	.08	-.01	**.81**	.07	.07	-.05	**.88**	.07	-.04	.04	**.83**	.08	.00
29	.07	.**81**	.00	.05	.06	**.77**	.05	.08	-.03	**.82**	.07	-.04	.05	**.80**	.10	.05
*PRES*																
2	-.02	.00	-.04	**.90**	.02	.07	-.09	**.86**	**.83**	-.01	-.03	-.05	**.80**	-.07	.03	-.12
6	.21	.09	-.07	**.69**	-.01	.20	-.02	**.77**	**.66**	.02	-.07	-.29	**.76**	.10	-.05	-.09
10	.08	-.02	-.02	**.89**	.03	-.06	-.02	**.96**	**.85**	-.03	-.04	-.10	**.98**	-.05	.01	.03
14	.20	**-.**01	-.01	**.77**	.09	.01	.01	**.84**	**.77**	.05	.06	-.10	**.68**	.13	.00	-.21
18	-.11	.01	.07	**.95**	-.02	-.08	.10	**.86**	**.91**	.01	.03	.06	**.79**	-.01	.10	-.08
22	-.04	-.01	.13	**.83**	.03	-.02	.16	**.72**	**.82**	.02	.16	.10	**.76**	.08	.13	.02
26	.06	.08	.00	**.81**	.03	.04	.00	**.85**	**.78**	.07	.01	-.07	**.66**	.07	-.01	-.25
*INT-GEN*																
3	**.77**	-.02	-.00	.11	**.77**	.15	-.08	.03	.21	.16	-.02	**-.56**	.13	.03	-.03	**-.73**
4	**.74**	.06	.04	.10	**.91**	-.03	-.03	.03	.06	.03	-.03	**-.85**	-.04	.12	.11	**-.76**
7	**.87**	-.00	-.01	-.04	**.79**	.04	.05	-.06	.13	.09	-.16	**-.65**	.24	.06	-.03	**-.64**
8	**.82**	.08	-.02	.03	**.89**	-.02	-.02	.05	-.03	-.03	.01	**-.95**	.07	.19	.07	**-.69**
11	**.90**	-.03	.04	-.06	**.88**	.05	.02	-.04	.11	.02	.09	**-.70**	-.00	.03	.10	**-.80**
12	**.85**	.02	.01	.04	**.85**	-.02	.01	.11	.07	.02	.06	**-.80**	.22	-.02	.04	**-.68**
15	**.72**	.04	.09	.02	**.74**	.06	-.00	.05	.07	.19	.19	**-.49**	.03	-.09	.05	**-.83**
16	**.85**	.07	.00	.02	**.82**	.03	.01	.10	.01	.09	.02	**-.82**	.20	.10	.11	**-.62**
27	**.43**	.13	**.**09	.29	.35	.12	.06	.35	.36	.18	.14	-.25	.17	.03	.06	**-.61**
*INT-ATH*																
19	.26	-.04	**.56**	.13	.30	.04	.**53**	.03	.19	.01	**.55**	-.17	-.01	.05	**.64**	-.30
20	.26	.02	**.40**	.17	.**61**	-.10	.26	.01	.06	.02	.34	**-.44**	.20	.10	**.42**	-.17
23	.01	-.02	**.93**	-.06	-.10	.12	**.87**	.11	.05	.01	**.92**	.03	.11	-.04	**.93**	.08
24	.03	.01	**.86**	-.02	.10	-.03	**.88**	-.05	-.08	-.04	**.75**	-.26	-.11	.03	**.89**	-.07
30	-.08	.08	**.74**	.06	-.01	.10	**.69**	.09	.09	.09	**.72**	.10	.04	.01	**.84**	.02

*Note.* Items are organized by the four subscales identified by Thompson et al., 2004. Specifically, *INFO* = Information; *PRESS* = Perceived Pressure; *GEN* = Thin-ideal Internalization; *ATH* = Athletic-ideal Internalization. Items with factor loadings ≥ .40 are in bold.

Finally, congruence coefficients were almost all in the very high range, which suggests that the factors could be considered equal across the four groups (see Table 2). The only value below .95 was the .91 for the congruence between the African and Asian American groups; of course, that value was still in the “fair similarity” range [46].

## Discussion

Overall, the SATAQ-3 exhibited strong psychometric properties in a sample of European American/White, African American/Black, Asian American, and Latina/Hispanic college women from the USA. Cronbach’s alpha values and MSAs were excellent for all four groups; patterns of eigenvalues indicated a four-factor model was appropriate for all groups; and factor analyses showed very similar patterns for European American/White, Latina, and Asian American women. These results are consistent with previous psychometric evaluations of the SATAQ-3 in female college samples [1,27].

That said, items 20 (“I compare my body to that of people in ‘good shape’”) and 27 (“I try to look like the people on TV”) did not load as expected for Latina and Asian American women and had very low loadings for the European and African American group. Specifically, item 20 loaded on a different subscale than expected for the Latina and Asian American groups (i.e., INT-GEN instead of INT-ATH, although just barely for the Asian American group) and item 27 did not load higher than .40 on any subscale. Additionally, both of those items had the lowest loadings on the intended subscale for both the European American and African American groups. Similar problems have been noted with item 20 in other studies [27,37,53], which lead some researchers to suggest removing the item [30,53]. Additionally, although less consistently documented in the literature, two recent studies reported low factor loadings for item 27 [30,53]. To complicate matters, however, item 27 is sometimes negatively worded and was in both studies noted above. Although the original validation study did not include negatively worded items, Thompson and colleagues [1] recommended reverse scoring eight items to test for response bias. Given difficulties reported in previous psychometric investigations with negatively worded items, we did not negatively word any items in this dataset, as suggested by Wheeler and colleagues [37]. However, the lack of consistency in wording could have influenced results for this item.

Additionally, when scored according to the factor structure established by Thompson and colleagues [1], it is interesting to note that African American/Black women reported significantly less perceived pressure, thin-ideal internalization, and athletic-ideal internalization than all other groups. Theoretically, this makes sense as mainstream American media present the ideal woman as thin, young, tall, and predominantly White. In contrast, the ideal female figure in African American/Black culture is curvy with medium sized breasts and large buttocks [54] and more value is placed on personal attributes (e.g., attitude, style) than solely on physical appearance [41,55,56]. Consequently, although women of all ethnic groups likely report some appearance-related pressure, African American/Black women may not internalize or feel as much pressure to attain the thin-ideal because it is not as relevant to the ethnic ideal of beauty.

Despite the importance of these findings, they should be considered in light of the study limitations. The sample comprised female college students who self-identified as belonging to one ethnic group, which may limit the generalizability of these findings to other populations (e.g., women of other ages, educational backgrounds, ethnicities). In addition, ethnic groups are highly heterogeneous: by generalizing results within each ethnic group, the study did not test the influence of various intra-group factors that may influence responses such as acculturation, generational status, or ethnic identity [20,39]. Future research examining the SATAQ-3 would benefit from examining ethnic subgroups (e.g., Korean American) and cultures (e.g., British samples). Finally, a new version of the SATAQ (i.e., the SATAQ-4) is currently being published [57]. Although we believe that the SATAQ-3 will continue to be widely used because of its strong psychometric properties, many linguistic translations, and subscale structure (e.g., the SATAQ-4 only has internalization and pressure subscales), it is a potential limitation. Of note, neither item 20 nor item 27 appears in the SATAQ-4.

Despite these limitations, these results lend support for the use of the SATAQ-3 and its subscales as proposed by Thompson and colleagues [1] among the ethnic groups that we studied. Future research on media internalization with multi-ethnic samples should examine internalization of other beauty ideals (e.g., a curvaceous-ideal). For example, despite having lower levels of thin-ideal internalization in this sample, research suggests that African American/Black women do internalize more ethnically-salient appearance ideals (e.g., a more pronounced buttocks, larger breasts, preferred skin color) and that a discrepancy between the curvaceous-ideal and one’s current body shape is associated with body image problems [54,58]. For example, Overstreet and colleagues [54] found that Black women preferred a curvier ideal than White women and that dissatisfaction with ones appearance in comparison to the curvaceous ideal predicted appearance-related concerns in Black women. In a sample of 124 African American college females, Falconer and Neville [59] found that greater satisfaction with one’s skin color was associated with more positive body image. Consequently, among African American/Black women, internalization of media depicting a curvaceous-ideal that highlight body areas more salient to African American/Black ideals of beauty (e.g., butt size, skin color, breast size) may be more salient to body image problems in this group. Future research should include a more detailed examination of this possibility and the role of perceived media pressure to attain different appearance ideals (e.g., curvaceous-ideal internalization) among ethnic groups who do not espouse ultra-thin beauty ideals for women.

## Conclusions

The purpose of the present study was to evaluate the score reliability and equivalence of factor structure of the SATAQ-3 in a sample of European American, African American, Asian American and Latina female college students. The results lend support to the use of the SATAQ-3 in these four ethnic groups, as evidenced by high score reliability and structure similarity of the four factors (i.e., awareness, perceived pressure, thin-ideal internalization and athletic-ideal internalization). Notably, questionnaire items 20 and 27 did not load consistently on the four-factor scale. In addition, African American participants’ scores on thin-ideal internalization and pressure were significantly lower than all other groups. Future directions include examination of the curvaceous, rather than the thin-ideal, as well as study of ethnic and cultural subgroups.

## Competing interests

The authors declare that they have no competing interests.

## Authors’ contributions

CSW designed the study, contributed to drafting of the manuscript and performed initial data analyses. DHG edited the manuscript and performed final data analyses. LMR helped write the manuscript and managed the dataset. All authors read and approved the final manuscript.
